# Cancer targeted drug delivery using active low-density lipoprotein nanoparticles encapsulated pyrimidines heterocyclic anticancer agents as microtubule inhibitors

**DOI:** 10.1080/10717544.2022.2117435

**Published:** 2022-08-26

**Authors:** Laila Jaragh-Alhadad, Haider Behbehani, Sadashiva Karnik

**Affiliations:** a Department of Chemistry, Faculty of Science, Kuwait University, Safat, Kuwait; b Cardiovascular and Metabolic Sciences Department, Cleveland Clinic Lerner Research Institute, Cleveland, OH, USA; c Cleveland Clinic Learner College of Medicine, Case Western Reserve University, Cleveland, OH, USA

**Keywords:** Nano-LDL particles, heterocyclic anticancer agents, endocytosis, targeted drug delivery, microtubules, MDA468, DU145

## Abstract

Recently, nanomedicine had the potential to increase the delivery of active compounds to specific cell sites. Nano-LDL particles are recognized as an excellent active nano-platform for cancer-targeted delivery. Loading of therapeutic agents into nano-LDL particles achieved by surface loading, core loading, and apolipoprotein-B100 interaction. Therefore, loading nano-LDL particles’ core with pyrimidine heterocyclic anticancer agents will increase cancer cytotoxic activity targeting tubulin protein. First, by mimicking the native LDL particle's metabolic pathway, and second the agent’s chemical functional groups like the native amino acids cytosine and thymine structures will not be recognized as a foreign entity from the cell’s immune system. Nano-LDL particles will internalize through LDL-receptors endocytosis and transport the anticancer agent into the middle of the cancer cell, reducing its side effects on other healthy cells. Generally, the data revealed that pyrimidine heterocyclic anticancer agents’ size is at the nano level. Agents’ morphological examination showed nanofibers, thin sheets, clusters, and rod-like structures. LDL particles’ size became bigger after loading with pyrimidine heterocyclic anticancer agents and ranged between 121.6 and 1045 nm. Then, particles were tested for their cytotoxicity against breast (MDA468) and prostate (DU145) cancer cell lines as surrogate models with dose-response study 10, 5, 1 µM. The IC_50_ values of the agents against DU145 and MDA468 possessed cell growth inhibition even at the 1 µM concentration ranges of 3.88 ± 1.05 µM and 3.39 ± 0.97 µM, respectively. In sum, nano-LDL particles proved their efficiency as active drug delivery vehicles to target tubulin in cancer cells.

## Introduction

Lipoproteins are a class of lipid and protein nano-particles that varies in their densities (Huang et al., 2015), their essential role is to transport fat within the body (Huang et al., 2015; Thaxton et al., 2016). Lipoproteins such as chylomicrons, very-low-density lipoproteins (VLDL), low-density lipoproteins (LDL), and high-density lipoproteins (HDL) have the same structure as triglycerides and cholesterol esters core, covered with phospholipids layer, and embedded apolipoproteins (Daniel et al., 2011; Thaxton et al., 2016; Browning et al., 2017). Many types of research showed the efficacy of drug-loaded into nano-particles in both in-vivo and in-vitro (Vitols et al., 1990; Vitols, 1991; Daniel et al., 2011; Huang et al., 2015; Thaxton et al., 2016; Browning et al., 2017; Mahmoudian et al., 2018). Due to this interest, many natural and synthetic nano-particle models are used and studied in biomedicine such as micelles (Torchilin, 2007), liposomes (Al-Jamal & Kostarelos, 2011), emulsions, and nano-LDL particles (Torchilin, 2007; Al-Jamal & Kostarelos, 2011; Mahmoud & Karen, 2012; Theresa & Pieter, 2013). Nanoparticles are used nowadays for the biomedical delivery applications of chemotherapeutics (Alhadad et al., 2020; Jaragh Alhadad, 2021), siRNAs, photosensitizers (Thaxton et al., 2016), and imaging contrast agents into different cells (Huang et al., 2015). Using the native lipoproteins metabolic pathway strategy as part of nano-LDL particles drug delivery, has many advantages because of their small size, biocompatibility, biodegradable (Thaxton et al., 2016; Mahmoudian et al., 2018), half-life, stability, and ability to bind to specific receptor LDL-receptor (Huang et al., 2015; Mahmoudian et al., 2018; Alhadad et al., 2020; Jaragh Alhadad, 2021).

**Figure 1.**
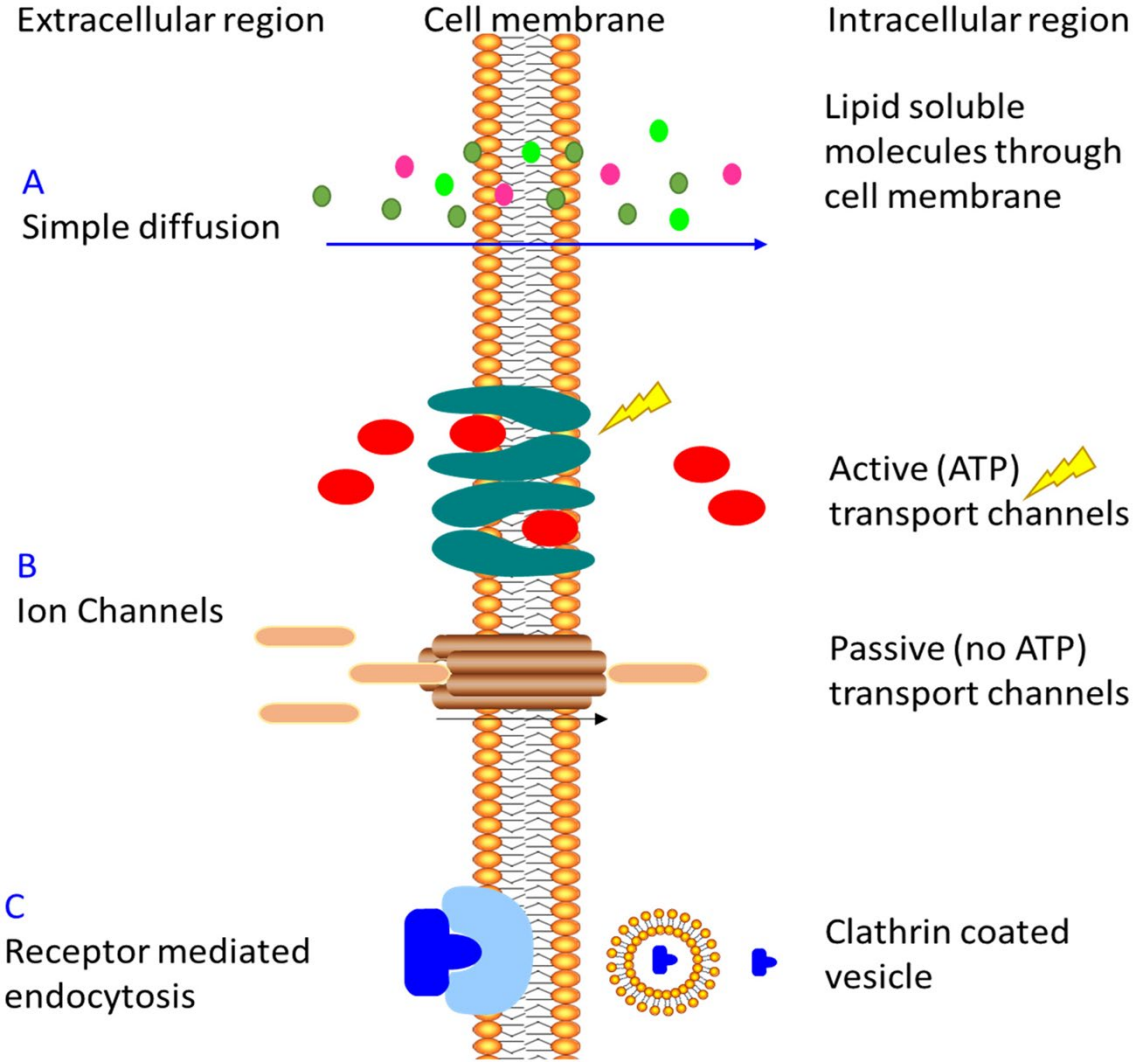
Cell uptake mechanisms.

Cells uptake molecules by three mechanisms. First, small molecules like water, oxygen, nitrogen, and carbon dioxide enter the cell by simple diffusion. Second, charged molecules like calcium, sodium, and glucose enter the cells by specific channels or transporters by active (with energy source-adenosine triphosphate ATP) or passive diffusion (without an energy source). Third, receptor-mediated endocytosis is associated with clathrin-coated vesicles as shown in Figure 1 (Bildstein et al., 2011; Kettler et al., 2014; Kuhn et al., 2014; Komin et al., 2017). Receptor-mediated endocytosis is an important uptake mechanism because it depends on the size of particles ranging from tens to hundreds of nanometers, and the high expression of the receptor in a specific cell type that benefits nanomedicine for gene and drug delivery (Zhong et al., 2002; Huajian et al., 2005; Pires et al., 2012). Generally, the liver synthesized VLDL that converts to IDL by lipase enzyme and then to LDL that circulates within the body to transport cholesterol cargo (Alhadad et al., 2020; Jaragh Alhadad, 2021). Circulating LDL particles are recognized by the cell through LDL-receptors bindings like key and lock through apolipoprotein B-100. Then, LDL enters the cell through a clathrin-coated vesicle, undergoes endosome and then lysosome degradation, leaving the cargo inside the cell and the LDL-receptor recycle back to the liver by HDL (Alhadad et al., 2020; Jaragh Alhadad, 2021). It is important to note that LDL-receptors are highly expressed in cancer cells more than in benign cells and this provides a good mechanism for internalization, drug concentration, and accumulation in the targeted cells and decreasing the side effects on the healthy cells (Maletínská et al., 2000; Pires et al., 2012; Radwan & Alanazi, 2014; Huang et al., 2015; Alhadad et al., 2020; Jaragh Alhadad, 2021).

Nano-LDL particles are used in cancer imageing (Gunasekera et al., 2009) and as drug carriers (Harisa & Alanazi, 2014; Alhadad et al., 2020; Jaragh Alhadad, 2021) such as treatment of cancer photodynamic therapy by delivery of hydrophobic and amphiphilic photosensitizers (Huntosova et al., 2012). Furthermore, Liping et al. designed and synthesized a novel naphthalocyanine photodynamic therapy agent and the results were promising in treating deeply seated tumors (Song et al., 2007). Moreover, a novel photosensitizer (TPA-DPPy) was encapsulated into LDL particles for the photodynamic killing of cancer cells that overexpress LDL receptors (LDLRs) A549 cell line, and the results were 88% efficiency (Chao et al., 2021). In 2020, DNA oligonucleotide aptamer, was synthesized to bind to LDL-R in both breast and liver cancers and the results showed high affinity and specificity (K_D_ = 19.6 nM) (Wang et al., 2020). Another research study used micro RNA-135a–VLDLR–p38 to target LDL-receptor in gallbladder cancer, the results showed cell proliferation reduction (Zhou et al., 2014). Other studies, prepared and characterized LDL-loaded cholesterol-siRNA coupled N-succinyl chitosan with doxorubicin and the data revealed significant inhibition of in-vitro tumor activity (Zhu et al., 2014). LDL-particles encapsulated anticancer agents targeting both cellular (HSP27) and receptor (HER2) proliferation proteins, and the results were excellent SKOV3 cell growth inhibition (Alhadad et al., 2020; Jaragh Alhadad, 2021). Further study, designed and synthesized 41 pyrimidine derivatives mimicking the natural amino acids’ natural structures cytosine, and thymine DNA building blocks to target triple-negative breast cancer, and the results were anti-proliferation and anti-metastatic activities (Yao et al., 2017).

Synthesizing anticancer agents that mimic the native amino-acid structures is a smart idea to target cancer without affecting immune system defenses. A study reported that small pyrimidine-containing molecules are considered anti-cancer agents (Abdellatif & Bakr, 2021), and other studies used pyrimidine as tyrosine kinase inhibitors (Adileh et al., 2021). Moreover, heterocyclic fused pyrimidines agents were synthesized as tubulin polymerization inhibitors to target the colchicine domain, the agents inhibited tumor growth in the A375 melanoma xenograft model, increased apoptosis, and disruption of tumor vasculature (Banerjee et al., 2018). Furthermore, new series of pyrimidine derivatives synthesized as inhibitors of tubulin polymerization and colchicine domain, the data revealed excellent potency in MCF7 cell death in the nano-molar levels (≤10 nM) (Islam et al., 2021). Generally, pyrimidines derivatives are used as anticancer agets (Nasser et al., 2016; El Sayed et al., 2020) targeting different cellular proteins such as tubulin (Sana et al., 2021), HSP90 (Davies et al., 2012), and vascular endothelial growth factor (VEGF) receptor (Aziz et al., 2016; Dawood et al., 2019).

**Figure 2.**
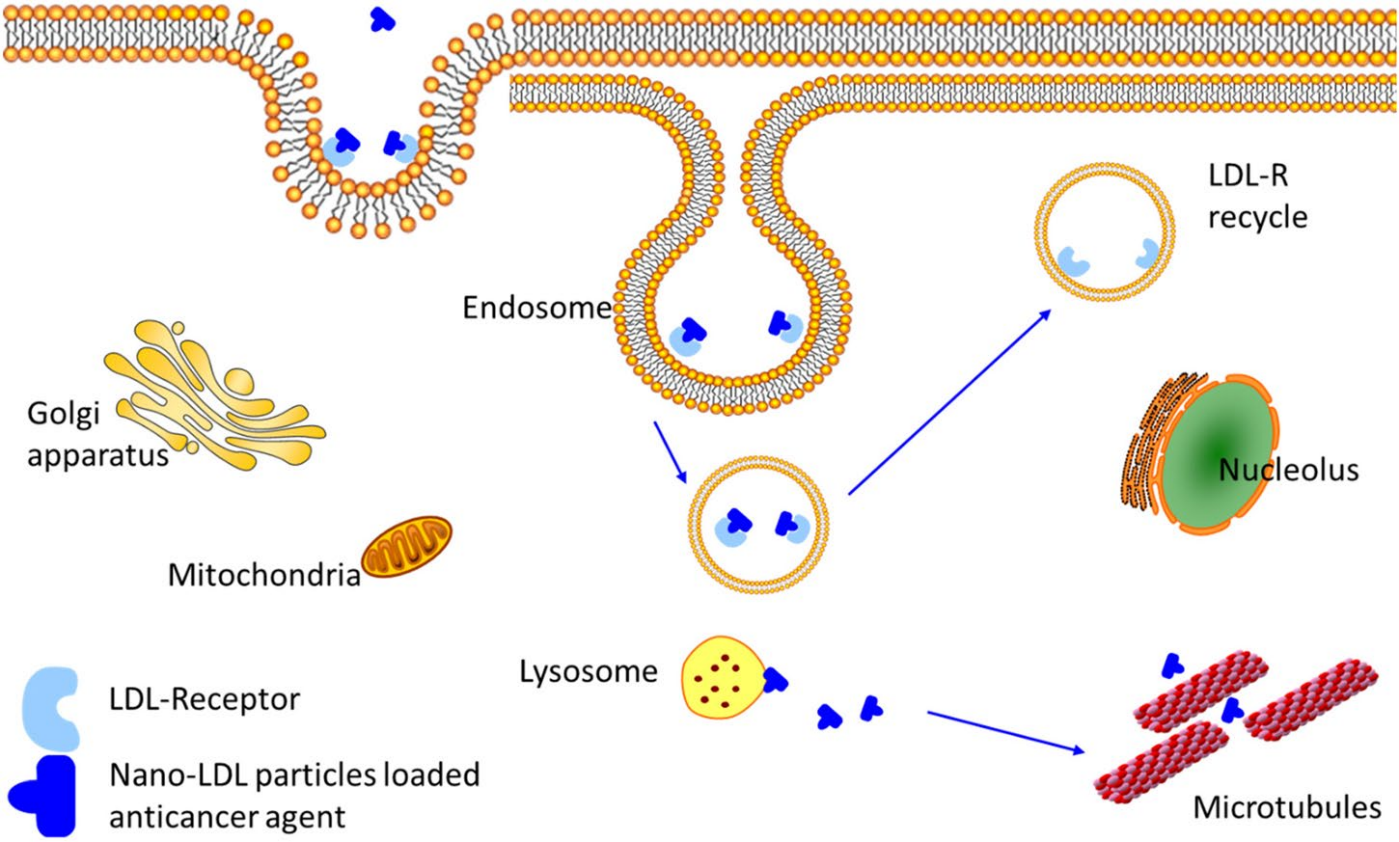
Targeting tubulin function by nano-LDL particles encapsulated with anticancer agents that binds to LDL-receptors to internalize into the cell.

Based on that, previously we synthesized pyrimidine heterocyclic anticancer agents their chemical functional groups mimic amino acids both cytosine and thymine chemical structure, applied structure-activity relationship in different positions with different moieties, and chemically and physically characterized the agents (Behbehani et al., 2012). The pyrimidine heterocyclic anticancer agent particles’ size and morphology were tested and then loaded into nano-LDL particles to ensure the effective cellular uptake by the cancer cell’s LDL-receptor and the bioactivity effect. Using two strategies to target proliferation protein microtubule (α, β tubulin) (Figure 2) which is an attractive strategy because microtubule is an important protein for cell division and mitosis. Its function and expression are elevated in cancer cells (Jaragh-Alhadad et al., 2022a; Laila et al., 2022).

## Materials and methods

### Chemicals

Anticancer agents were synthesized previously in our laboratory at Kuwait University, and (Behbehani et al., 2012) chemically and physically characterized at Kuwait University-Research Sector Project Unit (RSPU) laboratories. All the chemical reagents are commercially available with the analytical grades and are ready for direct use without any preparation from Merc and Sigma Aldrich.

Dulbecco Modified Eagle Media (DMEM), fetal bovine serum (FBS), phosphate buffer saline (PBS-pH 7.4), trypsin, L-glutathione, penicillin-streptomycin, and other supplements were supplied from the media core facility at Cleveland Clinic-Learner Research Institute. WST-1 kit was purchased from ABCAM. The LDL-depleted serum was purchased from Sigma Aldrich.

## Methods

### The morphology of the pyrimidine heterocyclic anticancer agents

The surface morphology of the agents was determined by taking five micrograms of the dry powder of the agents and placed on carbon tape on a stub. Then coated with platinum in sample coating machine before scanning electron microscope (SEM) reading. Five to sex SEM readings were taken at different magnifications X100, X250, X500, X1000, X2000, and X5000 (JEOL-EDS at 15.0 kV) system for each anticancer agent and photographed to visually display their stabilities.

### Agents’ particle size measurements

The synthesized pyrimidine heterocyclic anticancer agents were tested for dispersion with a variety of solvents such as water, dimethylformamide (DMF), ethanol, toluene, methanol, and dimethyl sulfoxide (DMSO). It was found that DMSO was the best, and the most suitable solvent for dispersion of the pyrimidine anticancer agents. The 100 µl of agents were dispersed in two ml of a suitable solvent (DMSO), and the size measurements were done using a zeta sizer at pH seven (Nano ZS-Malvern Paralytical Ltd, UK) at the Kuwait University-RSPU facility (Alhadad et al., 2020; Jaragh Alhadad, 2021).

### Preparation of loading pyrimidine heterocyclic anticancer agents into nano-LDL particles

The structure of the commercially available LDL-depleted serum is like the native LDL particles. Therefore, 50 µl of the nano-LDL particles were used for encapsulation with a five µl of heterocyclic anticancer agent with a ratio (5:1). The agents were mixed with nano-LDL particles by pipetting, vortexing, and sonicating using (Poly-Tron dispersing and mixing device made in Switzerland by Kinematica AG). Then, the samples were left to reform and reconstruct again overnight in a 4 °C fridge before measuring their particle sizes (Alhadad et al., 2020; Jaragh Alhadad, 2021).

### Nano-LDL particles’ size distribution after encapsulation

Briefly, after the agent’s encapsulation into nano-LDL particles, phosphate buffer saline (PBS-pH 7.4) was used as our blank and used for washing between each sample run in the zeta sizer device. First, ten µl of LDL was used as free control vehicle to measure the background in 1000 μl of PBS. This step was repeated for all LDL particles encapsulated with pyrimidine heterocyclic agents at pH seven and the size measurements were done using a zeta sizer (Nano ZS-Malvern Paralytical Ltd, UK) at Kuwait University-RSPU (Alhadad et al., 2020; Jaragh Alhadad, 2021).

### UV-VIS spectroscopy

Characterization of plain LDL particles and the encapsulated LDL particles with anticancer agents were done within a wavelength range of 300-600 nm by Flex Station-Molecular Devices at Lerner Research Institute at Cleveland Clinic. The UV-VIS absorption measurement for each sample was analyzed after centrifugation of the sample and the re-dispersion in distilled water (Abdellatif et al., 2021).

### Cell culture

A breast cancer (MDA468) and prostate cancer (DU145) cell line were purchased from the media core at Cleveland Clinic-Lerner Research Institute. Cells were maintained in DMEM media supplemented with 10% FBS, 1% penicillin/streptomycin, and 1% L-Glutathione, and incubated in humidified air with 5% CO_2_. FBS is inactivated at a 37 °C water bath for 30 minutes before use.

### Cells cytotoxicity assessment

The in-vitro cytotoxicity assay was carried out based on the manufacturer's protocol provided by WST-1 assay Kit (ab65473 Abcam, USA). Briefly, 5 × 10^4^ cell/well/ml from the breast and prostate cell lines were seeded on 96-well plates and incubated overnight. The cells were treated with 100 µl of various concentrations of heterocyclic anticancer agents and incubated for 48 hr. and then ten µl WST-1 reagent was added to each well. The absorbance was measured at a wavelength of 440 nm using a plate reader (SoftMax Pro 9.0 Flex Station-Molecular Devices) at Lerner Research Institute at Cleveland Clinic. The experiments were performed in quadruplicate concentrations (Alhadad et al., 2020; Jaragh Alhadad, 2021).

### Statistical analysis

Statistical analysis data were performed by SoftMax Pro and analyzed by GraphPad Prism software and the results normalized to controls by nonlinear regression analysis. The statistical data were presented as mean ± standard deviation (SD) from three independent tests. Significance was determined by the *P* values, where **P<0.001, ***P<0.001, ****P<0.0001.

## Results

### Anticancer agent’s morphology

Previously, the agents were fully investigated and characterized at RSPU laboratories (Behbehani et al., 2012), then particles’ morphology was tested at 15.0 kV, and the data showed nano-size particles, fibers, thin sheets, clusters, and rod-like structures (Figure 3). These nano-sized particles will benefit the loading process and make it easy to accumulate in the cancer cell. In addition, nanoparticles are excellent candidates for LDL encapsulating, penetrating, and transporting anticancer agents to cancer tumors and tissues due to their small size and binding affinity to LDL-receptor (Alhadad et al., 2020; Jaragh Alhadad, 2021). Generally, the nano size of the particles enhance the physiological and biological activities.

Figure 3.The SEM micrographs of the nano anticancer agents were observed and generally were smooth surfaces without holes or cracks viewed at different magnifications.

**Figure:**
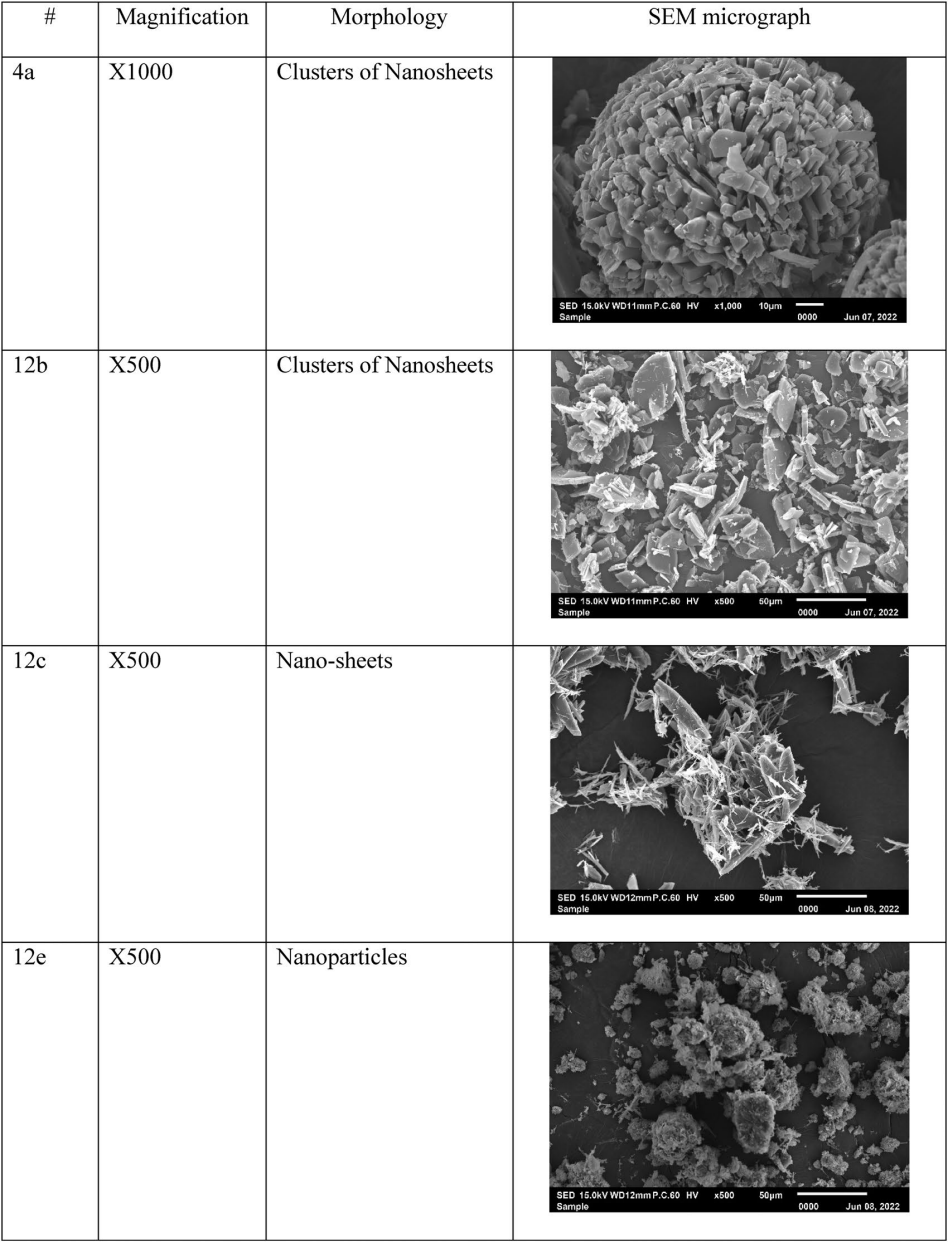

**Figure:**
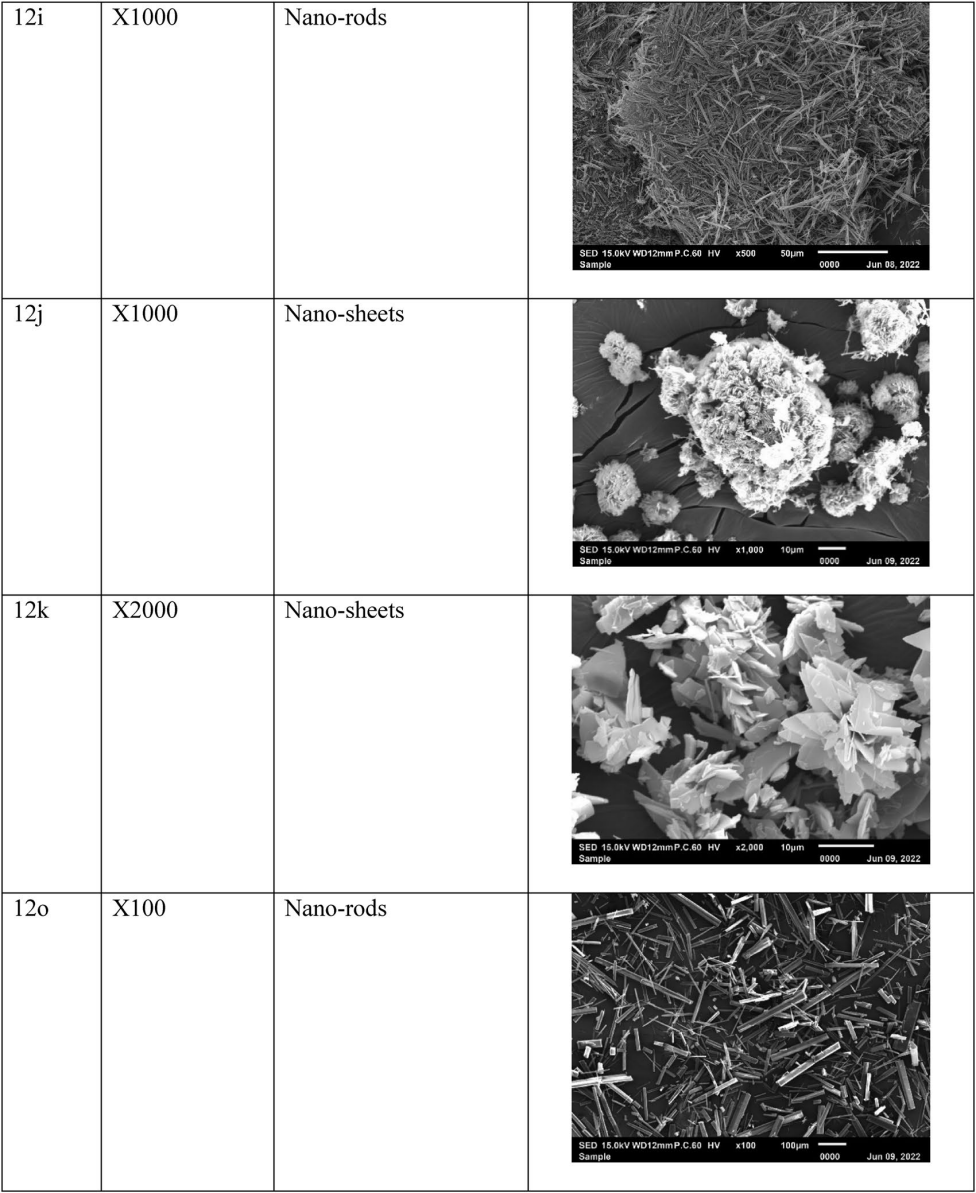

**Figure:**
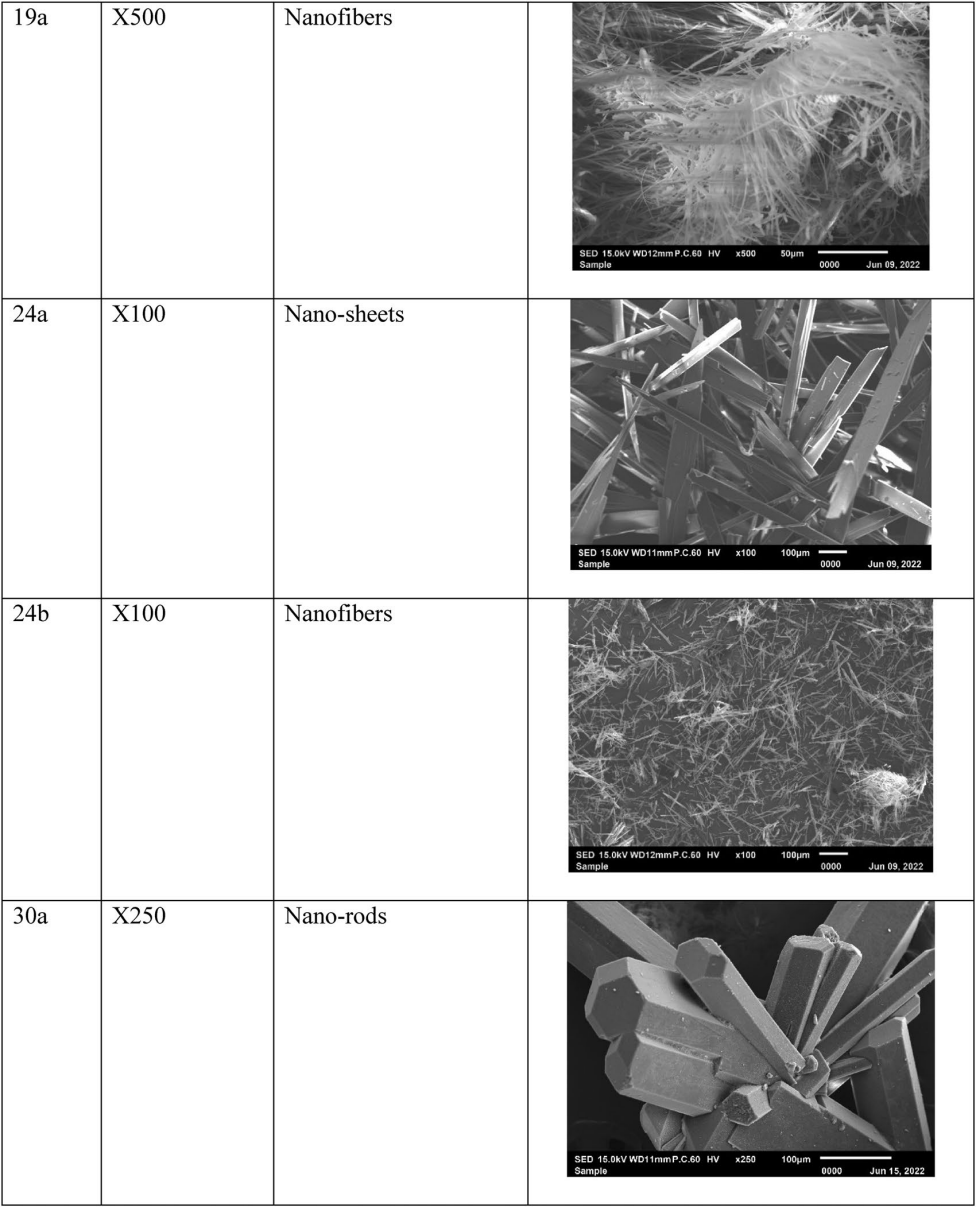

### Anticancer agent’s characterization

The agent's particle size measured with a zeta sizer revealed the agents had nano-size levels. It is important to note that agents with a nano-size level are better than the micro size level (Ignatova et al., 2011; Xing et al., 2011; Xiupeng et al., 2016; Kavinkumar et al., 2017; Abdellatif et al., 2021; Lara-Ochoa et al., 2021; Jaragh-Alhadad et al., 2022a). In addition, the agents showed different morphologies such as a rod-like structure (Xiupeng et al., 2016) and nano-fibers (Ignatova et al., 2011), that will disrupt the microtubule stability, and the small nanoparticles like nanoclusters and nanoparticles can disrupt α,β tubulin subunits (Xing et al., 2011; Kavinkumar et al., 2017). In sum, studies proved that nanoparticles showed anticancer activities (Alhadad et al., 2020; Jaragh Alhadad, 2021; Lara-Ochoa et al., 2021; Jaragh-Alhadad et al., 2022a).

### Agents’ size distribution before and after encapsulation with nano-LDL particles

**Figure 4.**
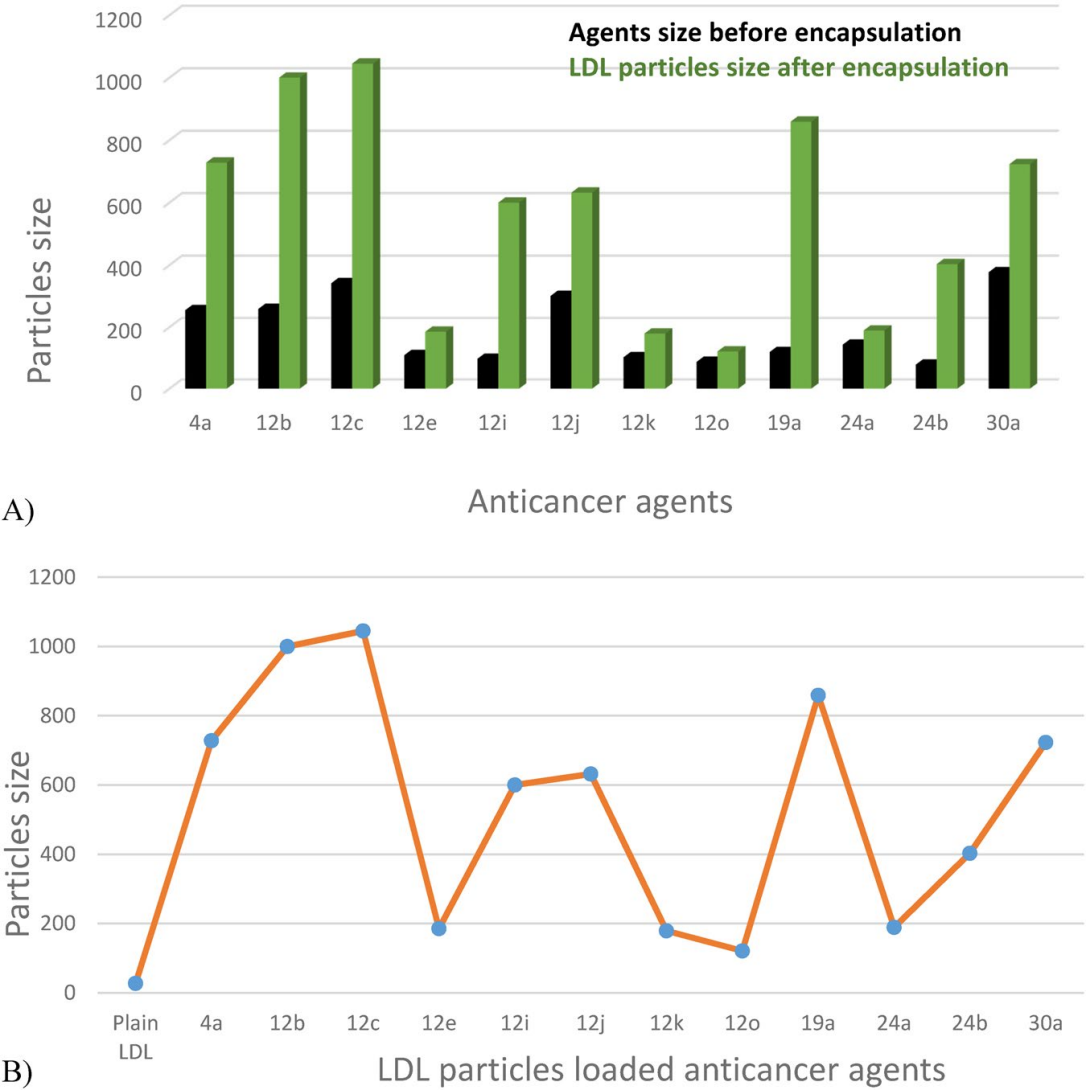
A) Pyrimidine heterocyclic anticancer agents’ particle sizes increased after LDL particles encapsulation. B) LDL particles encapsulated in anticancer agents 1-12 became bigger compared to the plain LDL particles’ size.

Pyrimidine heterocyclic anticancer agent’s particle size was dispersed in DMSO solvent, and the particles’ size was measured before and after encapsulation into LDL particles, the results were summarized in Table 1. The data revealed that the pyrimidine anticancer agents were in the nano-molar range between 78 nm to 377 nm before LDL particles encapsulation and reached between 121 nm to 1045 nm after LDL particles encapsulation (Figure 4A). Agent 4a particle size was 255 nm and became 728 nm after LDL particles encapsulation because the agent exhibited clusters morphology. In addition, agent 12 b particle size was 259 nm and became 1000 nm, and agent 12c particle size was 342 nm and became 1045 nm (Agents 12 b and 12c showed the biggest nano-LDL particles because of the clusters of the nano-sheets morphologies, respectively). Furthermore, agent 12e particle size was 109 nm and became 185 nm because it exhibited nano-particles morphology. Also, agent 12i particle size was 97 nm and jumped to 600 nm size because the agent had nano-rods structures. Moreover, agent 12j particle size was 301 nm and became 632 nm, agent 12k particle size was 102 nm and became 179 nm and this was due to the nano-sheets particles’ morphology. Agent 12o particle size was 87 nm and became 121 nm because of the nano-rods morphology. Further, agent 19a particle size was 119 nm and jumped to 859 nm because it exhibited nanofiber morphology. Agent 24a particle size was 143 nm and became 189 nm because of the nano-sheet morphology while agent 24 b particle size was 78 nm and jumped to 403 nm and this was due to the nano-fibers morphology. Agent 30a particle size was 377 nm and reached 723 nm because of the nano-rods morphology. Generally, LDL particles became bigger after encapsulation with the anticancer agents based on the size of the plain LDL (Figure 4B) (Abdellatif et al., 2016; Abdellatif, 2020; Abdellatif et al., 2021; Jaragh-Alhadad et al., 2022b).

**Table 1.** LDL particles size distribution.

Agent	Agents’ particle size before encapsulation nm	LDL particles size after encapsulation nm
Plain LDL	–	27.45
4a	255.0	728.5
12b	259.3	1000
12c	342.1	1045
12e	109.3	185.3
12i	97.65	600.7
12j	301.6	632.1
12k	102.8	179.0
12o	87.07	121.6
19a	119.4	859.8
24a	143.8	189.1
24b	78.82	403.1
30a	377.8	723.4

Plain LDL was used as our control vehicle.

In-depth, the commercially available LDL was used for anticancer encapsulation, which starts with simple pipetting, mixing, and vortexing. This was followed by sonication of the sample using Poly-Tron dispersion and a mixing device to break down the large LDL particles into small LDL particles. Samples were left in a 4 °C fridge overnight to give the small LDL particles the time to reform, and rap around the anticancer agents. This will form anticancer agents coated with LDL particles and each sample will have a specific LDL particle size based on its nano size and morphology either rod, fibers, clusters, or nanoparticles structures. Increasing the LDL particles size after encapsulation indicates the successful loading and encapsulation of the anticancer agents into the nano LDL particles core (Alhadad et al., 2020; Jaragh Alhadad, 2021; Jaragh-Alhadad et al., 2022b).

### LDL particles characterization

**Figure 5.**
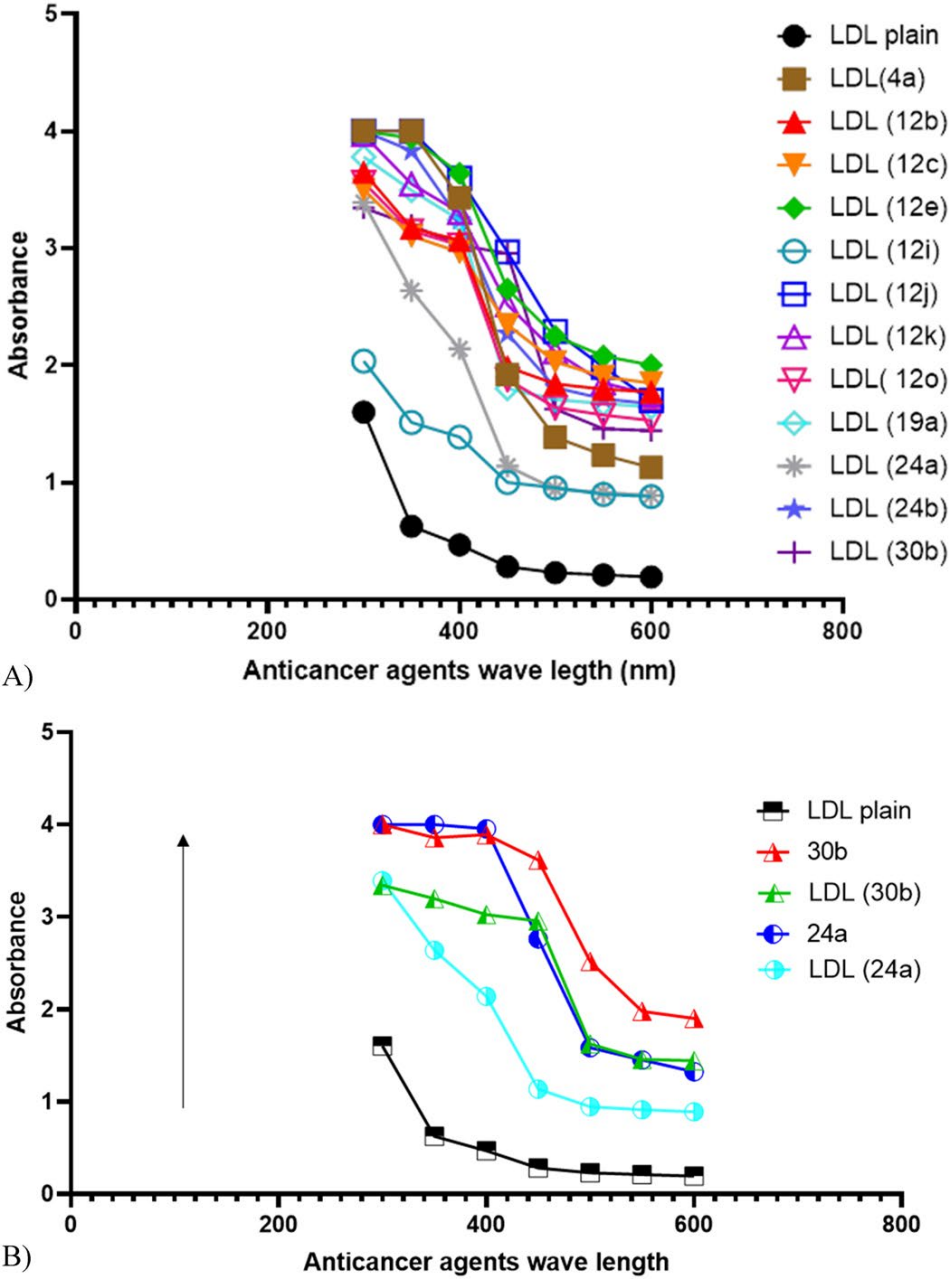
A) Plain LDL particles have absorbance reading in the visible region and all LDL encapsulated anticancer agents absorbance shifted to higher levels which means the agents were embedded in the LDL particles. B) LDL particles encapsulated agents 24a and 30 b has high absorbance reading more than the control and lower than the anticancer agents before encapsulation.

The UV-VIS spectra indicated that plain LDL particles showed a strong absorption of the electromagnetic waves in the visible region. The UV-VIS spectroscopy recorded wavelengths at 300, 350, 400, 450, 500, 550, and 600 nm (Abdellatif et al., 2016, 2021). The data showed a peak for plain LDL particles and shifted peaks to higher absorbencies for all LDL particles encapsulated pyrimidine heterocyclic anticancer agents because the agents were embedded in the LDL particles. In addition, the presence of one absorption peak for each sample indicated the symmetrical geometry and formulation for all LDL particles encapsulated with the agents (Abdellatif, 2020; Abdellatif et al., 2021). The shift observed in LDL particles encapsulated with pyrimidine heterocyclic anticancer agents could be attributed to the difference in the agent's shape and size distribution as shown in Figure 5A (Pal et al., 2016; Lewandowska & Kalinowska, 2020; Abdellatif et al., 2021). Furthermore, randomly, agents 24a and agent 30 b selected to read their absorbance before and after encapsulation compared to plain LDL as a control. The results showed LDL particles encapsulated with agent 24a has high absorbance reading more than the control and lower than agent 24a before encapsulation because the particles were encapsulated with nano sheets like structure lead to heavy particles and then lower absorbance. Furthermore, LDL particles encapsulated with agent 30 b showed similar results, it has higher absorbance reading more than the control and lower absorbance reading than the agent before encapsulation because the particles were encapsulated with nano-rods like structures as shown in Figure 5B.

### In-vitro cytotoxicity assessments

**Figure 6.**
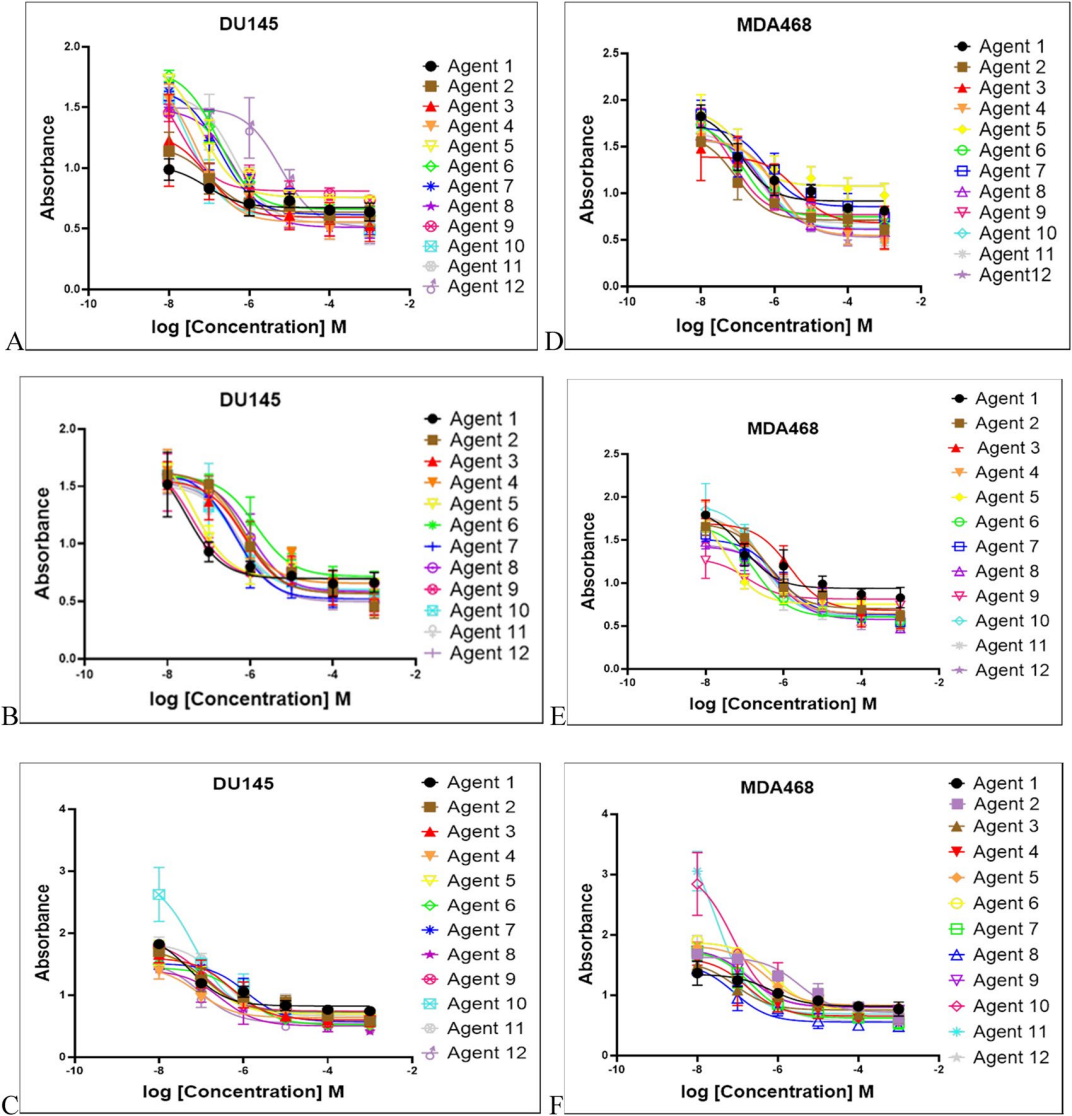
Anticancer agents treated with prostate cancer cell line (DU145) showed cell growth inhibition at 1 (A), 5 (B), and 10 µM (C). While anticancer agents treated with breast cancer cell line (MDA468) showed cell growth inhibition at 1 (D), 5 (E), and 10 µM (F) figures. All agents showed the ability to reduce cancer cell growth (DU145 & MDA468 cell lines).

**Figure 7.**
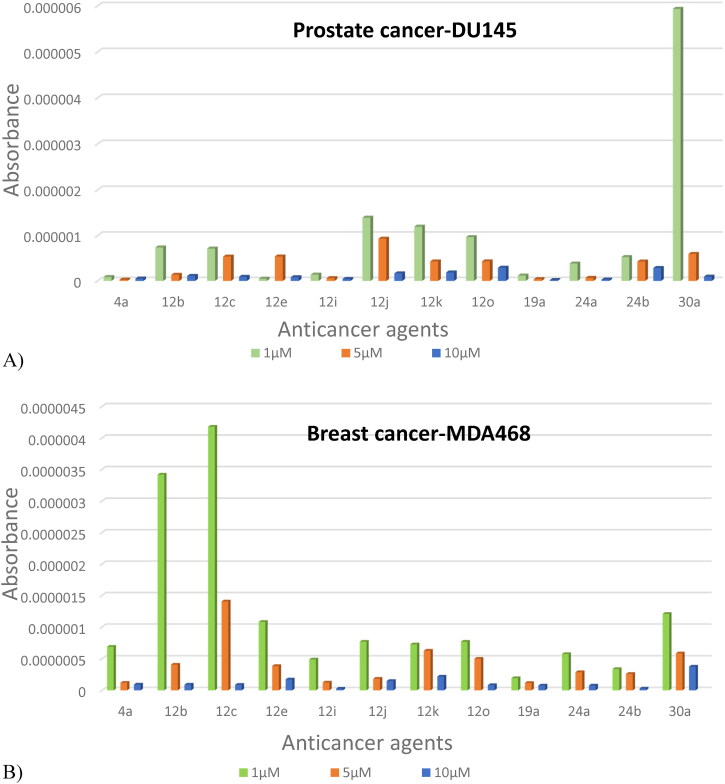
LDL particles encapsulated anticancer agents targeted DU145 and MDA468 cell lines at 10 µM> 5 µM> 1 µM.

Any drug delivery strategy starts with the agents’ stability both at 37 °C for treatment and at 4 °C for storage which proven previously (Abdellatif et al., 2021; Jaragh-Alhadad et al., 2022b). Several studies proved that LDL particles were stable until three months (Alhadad et al., 2020; Jaragh Alhadad, 2021; Jaragh-Alhadad et al., 2022b) and Abdellatif et al. confirmed that silver nan-particles physical stability reached up to three months at the following temperatures 25 ± 0.5 °C and 4.0 ± 0.5 °C (Abdellatif et al., 2021). Another study, in 2021 encapsulated anticancer agents into nano carbon core and the results prove the nanoparticles stability within two months and enabling the efficient tumor accumolation (Zhang et al., 2021). Additionally, studies proved that both DU145 (Sangrajrang et al., 1998; Edmondson et al., 2016) and MDA468 (Jones et al., 2014; Ibrahim et al., 2018) cell lines express tubulin protein. Furthermore, research studies showed that LDL-receptors are highly expressed in cancer cells more than in healthy cells (Song et al., 2009; Alhadad et al., 2020; Jaragh Alhadad, 2021) and induce cancer cell proliferation (Antalis et al., 2010; Gallagher et al., 2017), metastasis, and angiogenesis (Lu et al., 2017; Zhang et al., 2021; Tsumita et al., 2022). Based on that, pyrimidine heterocyclic anticancer agents were tested for the cytotoxicity effects with 48 hr. dose-dependent treatments after the one-month LDL-particles encapsulation. The IC_50_ data were collected and reported in Table 2 using ten µM, five µM, and one µM concentrations against DU145 and MDA468 cell lines as shown in Figure 6A–F. The results possessed cancer cell growth inhibition in both tested cell lines even at one µM concentration. In our previous project, we proved that plain LDL particles treated with cancer cells do not affect cell growth (Jaragh-Alhadad et al., 2022b). DU145 and MDA468 Cells treated with ten µM, concentration showed IC_50_ values ranging between 1.11–5.94 µM, and 1.21–5.05 µM, respectively. Also, DU145 and MDA468 cells treated with five µM, concentration showed IC_50_ values ranging between 1.34–5.95 µM, and 1.74–7.68 µM, respectively. Furthermore, DU145 and MDA468 cells treated with one µM, concentration showed IC_50_ values ranging between 3.88–9.66 µM, and 3.39–9.36 µM, respectively. The most potent agent that targeted prostate cancer cells was agent 19a while 24 b was the most potent agent to target breast cancer cells. Overall, active LDL particles encapsulated pyrimidine heterocyclic anticancer agents targeted both cell lines with similar potency at 10 µM> 5 µM> 1 µM as shown in Figure 7. Mimicking pyrimidine's native amino acid structures benefits cancer treatment and causes cell growth reduction (Yao et al., 2017; Islam et al., 2021).

**Table 2.** Breast and prostate cancer cell lines are treated in a dose-dependent manner.

DU145 prostate cells	IC_50_ values, one µM	IC_50_ values, five µM	IC_50_ values, ten µM
Agent 4a	8.59 ± 0.34	5.24 ± 1.18	2.84 ± 1.10
12b	7.38 ± 1.05	1.34 ± 1.01	1.11 ± 0.54
12c	9.13 ± 0.69	7.14 ± 0.98	5.36 ± 1.01
12e	8.19 ± 0.82	5.40 ± 0.97	4.92 ± 1.21
12i	6.17 ± 1.12	4.36 ± 1.18	1.40 ± 1.03
12j	9.31 ± 0.92	1.67 ± 1.14	1.40 ± 0.87
12k	4.29 ± 1.09	1.87 ± 1.04	1.20 ± 0.94
12o	9.66 ± 0.99	4.32 ± 0.97	2.91 ± 0.89
19a	3.88 ± 1.05	1.78 ± 1.01	1.13 ± 1.12
24a	6.71 ± 2.16	3.83 ± 0.98	3.07 ± 1.28
24b	5.26 ± 0.92	4.28 ± 1.07	2.85 ± 1.17
30a	9.54 ± 1.04	5.95 ± 1.04	5.94 ± 0.98
MDA468 Breast cells	IC_50_ values, one mM	IC_50_ values, five mM	IC_50_ values, ten mM
Agent 4a	9.36 ± 0.92	6.88 ± 0.53	1.21 ± 0.96
12b	9.11 ± 0.92	4.08 ± 0.99	3.42 ± 0.91
12c	8.89 ± 0.81	4.18 ± 0.71	1.41 ± 1.01
12e	3.86 ± 1.11	1.74 ± 0.96	1.08 ± 1.03
12i	4.91 ± 0.98	2.75 ± 1.27	1.26 ± 0.82
12j	7.68 ± 1.13	1.85 ± 1.07	1.49 ± 1.02
12k	7.26 ± 0.87	6.28 ± 0.85	2.19 ± 1.12
12o	8.26 ± 0.98	7.68 ± 0.84	5.01 ± 0.98
19a	7.66 ± 1.09	1.94 ± 0.96	1.19 ± 0.47
24a	7.78 ± 2.27	5.77 ± 0.96	2.88 ± 1.30
24b	3.39 ± 0.97	2.90 ± 1.17	2.62 ± 1.18
30a	5.87 ± 0.82	3.76 ± 1.12	1.21 ± 1.04

DU145 cell line: ****P value <0.0001 for all agents except, agent 4a and 12 b, ***P value <0.001, and agent 12c the **P value <0.001.

MDA468 cell line: ****P value <0.0001 for all agents except agent 12i, ***P value <0.001.

In addition, it is worth to be noted that based on the structures of the synthesized anticancer agents, loading and encapsulation helped with the internalization process because of the electrostatic, hydrophobic interactions, hydrogen bonding donor and acceptor that helps to increase the biological activities (Behbehani et al., 2012). Also, an increase of the drug concentration to 10 µM will cause more cell death than 1 µM and this suggest the strength of the interactions of the anticancer agents with the cellular tubulin protein after internalization process. Further, incubation time of LDL particles encapsulated anticancer agents with the tested cell lines at 48 h showed potent activities more than 24 h treatments (Wang et al., 2019; Jaragh-Alhadad et al., 2022b).

In 2022 Jaragh-Alhadad et al., proved that LDL nanoparticles encapsulated with thiosemicarbazone anticancer agents, considered an efficient vehicle for drug delivery by core loading method causing apoptosis against MCF7, A549, and C42 cell lines with IC_50_ values 1.18–6.61 µM, 1.17–9.66 µM, and 1.01–6.62 µM, respectively (Jaragh-Alhadad et al., 2022b). Huntosova et al., used HYP loaded into LDL particles core coated with dextran, internalized by LDL-receptor and increased the cellular uptake by U-87 MG cells (Huntosova et al., 2012). The flow cytometry experiment proved the quantitative cellular uptake for LDL particles at 37° C by endocytosis. In addition, to the fluorescence imaging which was used as an endocytosis marker (Huntosova et al., 2012). Further, study used LDL nanoparticles loaded with naphthalocyanine photosensitizer for photodynamic therapy (Song et al., 2007). The UV spectrum of the LDL particles before and after reconstitution was the same which proved the lipid core loading strategy. Also, the size of the nanoparticles was in the range of the native LDL particles (Song et al., 2007). Another research study stated that lipoproteins are promising drug carriers targeting cancer tissues (Tian et al., 2017). Moreover, LDL encapsulated doxorubicin nanoparticles endocytosis to tumor cell and the results revealed apoptosis by blocking AKT/mTOR signaling pathway (Kader et al., 1998). Additionally, LDL encapsulated sorafenib and dihydroartemisinin showed remarkable cell decrease in the cell viability study with *P* value: ***P<0.001. Therefore, our agents proved to be potent anticancer agents compared to all these findings (Wang et al., 2019).

## Discussion

LDL particles proved to be an active platform for diagnosis (Huntosova et al., 2012; Harisa & Alanazi, 2014) and cancer treatments (Alhadad et al., 2020; Jaragh Alhadad, 2021; Jaragh-Alhadad et al., 2022a, 2022b) because of their physicochemical and biological properties (Abdellatif et al., 2016; Abdellatif, 2020; Abdellatif et al., 2021; Jaragh-Alhadad et al., 2022b) which increases their fields of applications. In this study, we applied a lot of strategies to reduce cancer cell growth in the tested cell lines. First, mimics the native LDL particle's metabolic pathway to target cellular tubulin (Alhadad et al., 2020; Jaragh Alhadad, 2021; Jaragh-Alhadad et al., 2022b). In 2020, Alhadad et al., proved that low density lipoprotein encapsulated anticancer dual HSP27 and HER2 inhibitor targeted ovarian cancer and caused cell death (Alhadad et al., 2020). Furthermore, in 2021 Alhadad proved that LDL is active platform for anticancer drug delivery (Jaragh Alhadad, 2021). Second, the chemical functional groups in the agent's skeleton are like the functional groups of the amino acids’ cytosine and thymine DNA building blocks. A lot of research studies stated that pyrimidines derivatives are considered anticancer agents (Nasser et al., 2016; Yao et al., 2017; El Sayed et al., 2020) especially targeting the aggressive triple negative breast cancer (Yao et al., 2017).

Third, our anticancer agents’ sizes were proved to be in the nano levels based on zeta data and scanning electron microscope data proved their morphologies. Agents’ morphology disrupts the microtubule's stability (Tsumita et al., 2022) and the nano-size particles increase the cellular uptake. A study, stated that rode shape anticancer agents increase the cellular uptake in-vitro and stimulate anticancer activity in-vivo (Xiupeng et al., 2016). Furthermore, a study proved that DOX-containing nano-fibers agent possessed good anti-proliferation activity against MCF7 cell line (Ignatova et al., 2011). In addition, the graphene oxide nanosheets showed high toxicity against the lung cancer A549 cell line (Kavinkumar et al., 2017). Moreover, the clusters of magnetic platinum anticancer nano-drugs displayed excellent dimensional uniformity and exert high cytotoxicity toward human cervical cancer and human hepatocarcinoma (Xing et al., 2011). Recently, a study proved that CuS nanoparticles wrapped inside carbon core had superior advantages in stability, tumor accumulation through enhanced permeability, and retention effect (Zhang et al., 2021). Generally, these different structures of the nanoparticles are disrupting the tubulin polymerization/depolymerization process of the dynamic, structural microtubule and disrupt cancer cell division which is followed by apoptosis (Lara-Ochoa et al., 2021; Jaragh-Alhadad et al., 2022b). More recently, in 2022, nano-drug (RCH) co-assemble with hemin, celecoxib (NSID), and roscovitine (cyclin dependent kinase 5 inhibitor) was studied in-vitro and in-vivo targeting ferroptosis (the non-apoptotic cell death pathway), and the results showed immune response induction, glutathione peroxidase pathway destruction, and ferroptosis inducing immunotherapeutic efficacy (Zhang et al., 2022). These nano-size particles benefit drug delivery (Abdellatif et al., 2021; Jaragh-Alhadad et al., 2022b) and lead to higher protein absorption (Lara-Ochoa et al., 2021). Also, proved the importance of our anticancer agent's size and shape which increase the interactions with the structural protein microtubules, and then disrupt the cell division and cause cancer cell death (Banerjee et al., 2018).

**Figure 8.**
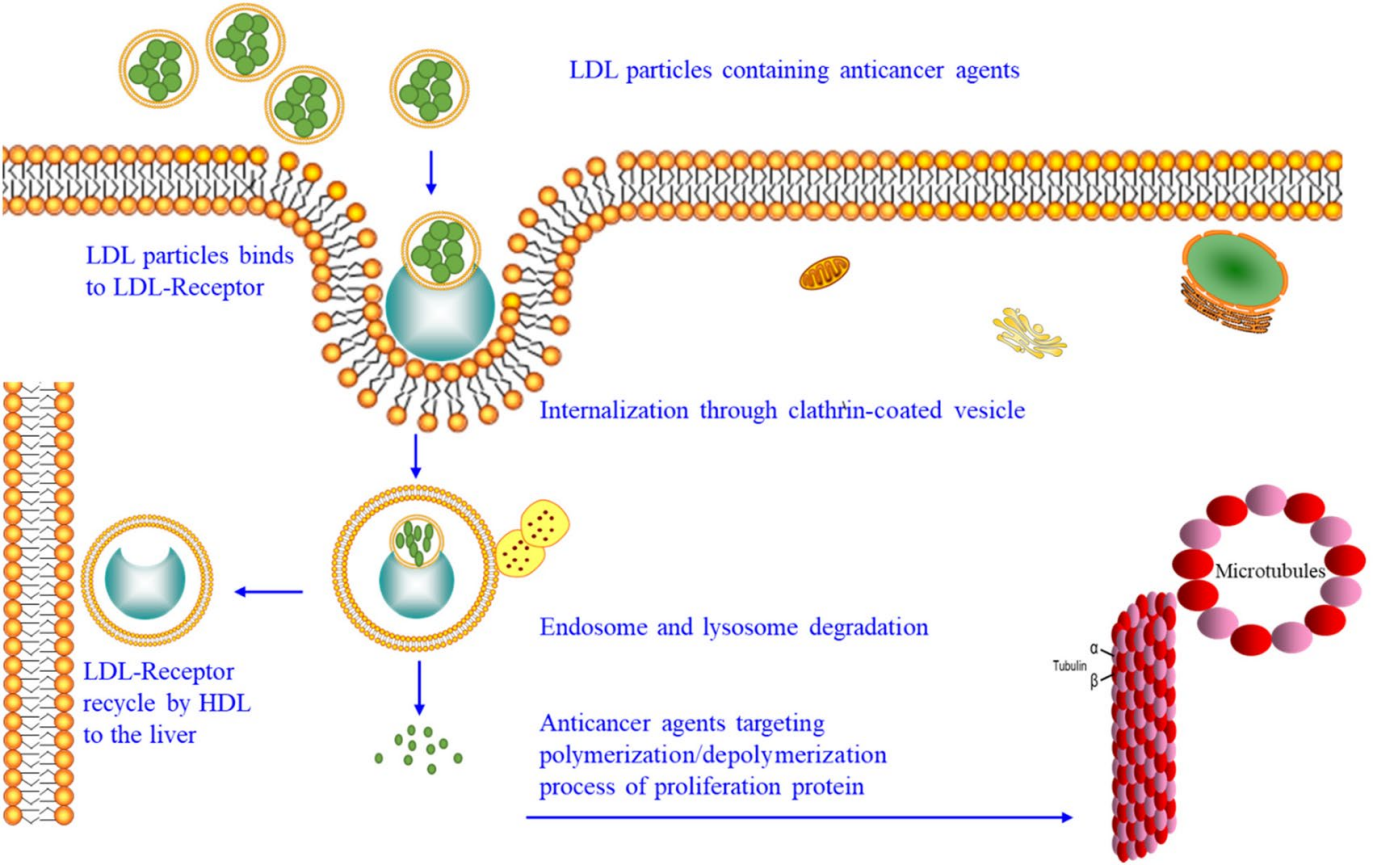
Schematic representation of LDL particles carrying anticancer agents, internalizing into the cell, and targeting microtubules stability.

Fourth, low density lipoprotein is naturally occurring nanoparticle that is biocompatible, biodegradable, and non-immunogenic (Song et al., 2007; Tian et al., 2017; Jaragh-Alhadad et al., 2022b; Laila et al., 2022). based on that nano-LDL particles were encapsulated with pyrimidine heterocyclic anticancer agents proved their stability and encapsulation based on UV-vis absorption reading. Our nano-LDL particles then bind to LDL-receptor which is expressed in cancer cells more than in healthy cells. Therefore, when loading anticancer agent into LDL particles will increase the binding to LDL-receptor which lead to endocytosis targeting microtubules as explained in Figure 8 and cause cancer cell death which is proved from the in-vitro assays’ data (Jaragh-Alhadad et al., 2022b). Zhang et al., in 2021 proved that anticancer agent coated carbon nanoparticles showed potent photodynamic performance for cancer therapy (Edmondson et al., 2016). All these smart strategies increase cancer cell death and apoptosis (Banerjee et al., 2018; Jaragh-Alhadad et al., 2022b; Laila et al., 2022). Alhadad et al. proved LDL particles encapsulation strategy as a potent active drug delivery against different human cancers such as ovarian, breast, lung, and prostate (Alhadad et al., 2020; Jaragh Alhadad, 2021; Jaragh-Alhadad et al., 2022b). In 2021, Abdellatif et al. proved that nanoparticles can deliver anticancer drugs to specific tissues and to enhance the tumor-killing effects of chemotherapeutic agents (Abdellatif et al., 2021) and studies showed nanoparticles applications due to their easy surface functionalization, stability (Kader et al., 1998; Zhang et al., 2021), conjugation (Tian et al., 2017), interactions, and biocompatibility can target tubulin (Abdellatif et al., 2016; Abdellatif, 2020). In short, nano-LDL particles encapsulated anticancer agents benefit cancer treatments for targeted delivery of diagnostic and therapeutic to LDL-receptor positive cancers (Song et al., 2007).

## Conclusion

These days there is a major need for a safe and efficient vehicle for cancer drug delivery. In this study, nano-LDL particles are used as an active drug delivery platform to transport anticancer agents into the middle of cancer cells mimicking the native LDL metabolic pathway, mimicking the amino acids functional groups, the shape of agents, the size of the agents before and after encapsulation with nano-LDL particles, then LDL particles internalization through highly expressing LDL-receptors cancer cells targeting microtubule function. The results revealed agents with nano size and showed excellent loading into nano-LDL particles. IC_50_ data showed potent cell death in dose-dependent studies. In sum, using multi strategies for drug targeting increase the cellular uptake and accumulation of the anticancer agents inside cancer cells and leads to potent biological effects.
